# Colon-targeting potential and metabolic effects of sodium butyrate-loaded CPC microspheres in type 2 diabetic mice

**DOI:** 10.3389/fnut.2026.1956044

**Published:** 2026-10-28

**Authors:** Qinqin Zhang, Gaiqi Yao

**Affiliations:** 1 Department of General Medicine, Peking University Third Hospital, Beijing, China; 2 Department of Critical Care Medicine, Peking University Third Hospital, Beijing, China

**Keywords:** colon-targeting potential, gut microbiota, metabolic health, short-chain fatty acids, sodium butyrate, type 2 diabetes mellitus

## Abstract

**Background:**

Oral sodium butyrate (SB) is rapidly absorbed in the proximal small intestine, limiting its colonic availability and local metabolic effects. We evaluated the *in vitro* release characteristics of chitosan-protocatechualdehyde/calcium alginate microspheres loaded with SB (CPC/SB), together with metabolic and microbial outcomes in mice with high-fat diet/streptozotocin-induced diabetes.

**Methods:**

CPC/SB release was measured in separate simulated gastric, intestinal, and colonic fluids. Thirty-five male C57BL/6J mice were assigned to seven groups: normal control and six diabetic groups receiving vehicle, unloaded CPC, free SB, CPC/SB, metformin, or metformin plus CPC/SB for 4 weeks (34 completed). Endpoints included gastrointestinal and fecal butyrate; blood glucose, homeostasis model assessment of insulin resistance (HOMA-IR), low-density lipoprotein cholesterol (LDL-C), and interleukin-6 (IL-6); and fecal microbiota assessed by 16S rRNA gene sequencing and PICRUSt2.

**Results:**

Relative butyrate release was 0.91%, 8.53%, and 94.17% in simulated gastric, intestinal, and colonic fluids, respectively. After CPC/SB administration, colonic butyrate exceeded gastric and small-intestinal levels, and fecal butyrate increased versus the model group (0.0523 ± 0.0121 vs. 0.0180 ± 0.0068 μg/mg; *p* < 0.0001). Random blood glucose decreased to 18.64 mmol/L at week 4 (*p* < 0.0001 vs. baseline and model). CPC/SB also reduced HOMA-IR (3.14 ± 0.43 vs. 4.81 ± 1.30; *p* = 0.046) and serum IL-6 (85.82 ± 1.90 vs. 93.55 ± 4.10 pg./mL; *p* = 0.008); LDL-C and α-diversity were unchanged. Exploratory 16S analyses suggested taxonomic and predicted pathway differences, but none survived Benjamini-Hochberg correction.

**Conclusion:**

At the tested dose, CPC/SB increased fecal butyrate, improved glycemic indices, and reduced serum IL-6 relative to the diabetic model group. SB, carrier, and delivery-related effects could not be separated, and *in vivo* targeting efficiency was not demonstrated.

## 1 Introduction

Type 2 diabetes mellitus (T2DM) is a chronic metabolic disorder characterized by insulin resistance and pancreatic β-cell dysfunction. Its global prevalence continues to rise and it has become a major public health burden (1). Although glucose-lowering agents such as metformin (MET), sulfonylureas, and sodium–glucose cotransporter-2 (SGLT-2) inhibitors are widely used in clinical practice, a considerable proportion of patients still fail to achieve glycemic targets, and long-term therapy is accompanied by limitations including gastrointestinal intolerance, hypoglycemia, and cardiovascular safety concerns, underscoring the need to develop novel interventional strategies.

In recent years, the pivotal role of the gut microbiota in the pathogenesis of metabolic diseases has become increasingly clear. Through metagenomic analysis, Qin et al. (2) first demonstrated that T2DM patients exhibit characteristic dysbiosis, mainly manifested as a reduced abundance of butyrate-producing bacteria (e.g., Roseburia and Faecalibacterium) and a relative enrichment of certain Bacteroides species that have previously been reported in association with T2DM, suggesting that these microbial disturbances are closely associated with the pathophysiology of T2DM. Subsequent studies have further shown that the gut microbiota is involved in host glucose and lipid metabolism through multiple pathways, including short-chain fatty acid metabolism, bile acid transformation, maintenance of intestinal barrier function, and regulation of systemic inflammation (3, 4).

Among various microbial metabolites, butyrate has attracted considerable attention because of its multi-target metabolic protective effects: it can act as a histone deacetylase inhibitor to regulate the expression of genes related to insulin signaling pathways (5), activate free fatty acid receptors (FFAR2/FFAR3) to promote glucagon-like peptide-1 (GLP-1) secretion (6, 7), enhance intestinal epithelial barrier integrity to reduce endotoxin translocation and systemic inflammation (8–10), and participate in energy metabolism and adipose tissue function regulation (11, 12). However, the clinical translation of butyrate faces a key bottleneck: orally administered SB is rapidly absorbed in the proximal small intestine, making it difficult to reach the colon at effective concentrations to exert local regulatory functions (11). Achieving colon-targeted delivery of butyrate has thus become a core scientific question for improving its therapeutic efficacy.

Chitosan (CS)–protocatechualdehyde (PA)/calcium alginate composite (CPC) microspheres loaded with sodium butyrate (SB), referred to as CPC/SB microspheres, were designed as a formulation with colon-targeting potential. The proposed structural basis involves Schiff-base condensation between PA and chitosan, Ca^2+^-mediated crosslinking of alginate, and electrostatic association of CS-PA with the alginate network. The design aims to limit premature gastric release and favor release under distal intestinal conditions. Chitosan itself has antimicrobial activity and has been reported to modulate gut microbiota composition (13, 14).

Based on the above background, this study evaluated the effects of CPC/SB microspheres on gut microbiota composition and metabolic outcomes in T2DM mice, with unloaded CPC and free sodium butyrate included as comparison groups. The effects of CPC/SB in combination with metformin were also examined to provide an experimental basis for further investigation of this formulation in T2DM.

## 2 Materials and methods

### 2.1 Experimental animals

All animal experiments in this study were approved by the Ethics Committee of the Animal Research Center of Peking University Third Hospital [ethics approval document number: (2025) Animal Ethics Review No. (143-02)]. The C57BL/6J mice used were purchased from the Department of Laboratory Animal Science, Peking University Health Science Center. The animals were housed in the surgical platform’s rodent temporary housing area of the Department of Laboratory Animal Science, Peking University Health Science Center.

### 2.2 Drugs and reagents

The sources of the main reagents and materials used are as follows. A high-fat diet providing 60% of energy from fat (catalog number 12492M) was purchased from Beijing Boaigang Biotechnology Co., Ltd. (Beijing, China); chitosan (catalog number C105802-25 g, medium viscosity, 200–400 mPa·s), sodium alginate (catalog number R129658, viscosity 200 ± 20 mPa·s), sodium butyrate (catalog number S102954, purity ≥ 98%), liquid paraffin (catalog number S59783), metformin hydrochloride (catalog number M107827, purity ≥ 97%), and regenerated cellulose dialysis bags (catalog number SP132594, molecular weight cutoff 3,500 Da) were purchased from Shanghai Yuanye Bio-Technology Co., Ltd. (Shanghai, China); streptozotocin (catalog number A152711), sodium citrate buffer (catalog number D100710, 0.1 mol/L, pH 4.5), protocatechualdehyde (catalog number A118859), Span 80 (catalog number A152651), and calcium chloride (catalog number A268031) were purchased from Shanghai Amole Biotechnology Co., Ltd. (Shanghai, China). Assay kits for mouse insulin (catalog number MK3315B) and IL-6 (catalog number MK5737A) were purchased from Jiangsu Meike Biotechnology Co., Ltd. (Zhangjiagang, Jiangsu, China), and the LDL-C assay kit (catalog number A113-1-1) was purchased from Nanjing Jiancheng Bioengineering Institute Co., Ltd. (Nanjing, China). Fecal genomic DNA was extracted using the QIAamp PowerFecal Pro DNA Kit (catalog number 51826; QIAGEN, Hilden, Germany). A mixed standard containing eight short-chain fatty acids and 2-ethylbutyric acid (internal standard; purity ≥ 99%) was purchased from Sigma-Aldrich (Merck KGaA, Darmstadt, Germany) for gas chromatography–mass spectrometry (GC–MS) analysis.

### 2.3 Preparation of CPC/SB microspheres

CPC/SB microspheres were prepared by a water-in-oil (W/O) microemulsion method combined with Ca^2+^-mediated ionic crosslinking of sodium alginate and electrostatic deposition of protocatechualdehyde-modified chitosan (CS-PA) onto the microsphere surface, as described below.

#### 2.3.1 Preparation of CS-PA

CS-PA was synthesized with minor modifications based on the method reported by Lv et al. (15). For each synthesis batch, 2.0 g of medium-viscosity CS was added to 100 mL of 1% (v/v) acetic acid solution in a 250-mL Erlenmeyer flask. The acetic acid solution was prepared by mixing 1 mL of glacial acetic acid with 99 mL of deionized water. The mixture was magnetically stirred until the chitosan had completely dissolved and a clear, slightly viscous solution was obtained. Subsequently, 0.1 g of PA was added, corresponding to a PA-to-CS mass ratio of 1:20. The reaction mixture was continuously stirred at room temperature for 6 h to allow Schiff-base condensation between the aldehyde groups of PA and the amino groups of CS.

After the reaction, the resulting solution was transferred into dialysis tubing with a molecular-weight cutoff of 3,500 Da and dialyzed against at least 2 L of deionized water for 5 days under gentle stirring to remove residual acetic acid, unreacted PA, and other low-molecular-weight impurities. The dialysis water was replaced every 24 h. After dialysis, the retained solution was collected, pre-frozen at −20 °C overnight, and subsequently lyophilized until a dry, porous solid was obtained. The freeze-dried CS-PA was ground into a powder, sealed, and stored until further use. Approximately 1.3 g of freeze-dried CS-PA was recovered from each synthesis batch.

#### 2.3.2 Preparation of sodium butyrate-loaded CPC/SB microspheres

CPC/SB microspheres were prepared using a W/O microemulsion method combined with Ca^2+^-mediated ionic crosslinking. For each preparation batch, 25 mg of SB was completely dissolved in 12.5 mL of 1 wt% sodium alginate solution to form the aqueous phase. Separately, 62.5 mL of liquid paraffin and 3.125 mL of Span 80 were added to a 250-mL beaker and mixed to form the oil phase. The oil phase was stirred and emulsified for 30 min. The SB-containing sodium alginate solution was then slowly added dropwise to the emulsified oil phase and stirred at 900 rpm for 30 min to form a W/O primary emulsion.

Subsequently, 6.125 mL of 0.5 wt% CS-PA solution was mixed with an equal volume of 10 wt% calcium chloride solution. The resulting CS-PA/CaCl₂ mixture was slowly added dropwise to the continuously stirred primary emulsion, and stirring was continued at 900 rpm for 3 h. During this process, Ca^2+^ interacted with the guluronic acid residues of sodium alginate to form an ionic “egg-box” crosslinked structure. Simultaneously, positively charged CS-PA was deposited onto the negatively charged calcium alginate surface through electrostatic interactions, thereby forming CPC/SB microspheres.

After completion of the reaction, the microspheres were collected by centrifugation at 4,000 rpm. The recovered product was washed sequentially with petroleum ether, absolute ethanol, and deionized water to remove residual liquid paraffin, surfactant, unreacted reagents, and other soluble impurities. The washed product was pre-frozen and lyophilized to obtain dry CPC/SB microsphere powder, which was stored at 4 °C until further use. The freeze-dried product recovered from one standard preparation batch weighed 34.62 mg.

#### 2.3.3 Preparation of unloaded CPC microspheres

Unloaded CPC microspheres were prepared using the same procedure described in Section 2.3.2, except that sodium butyrate was omitted from the sodium alginate aqueous phase. The resulting microspheres contained no sodium butyrate and were used as the carrier control.

#### 2.3.4 *In vitro* release assay

Ten milligrams of CPC/SB microspheres were separately incubated with shaking at 37 °C in 10 mL of simulated gastric fluid (SGF, pH 1.2), simulated intestinal fluid (SIF, pH 6.8), simulated colonic fluid (SCF, pH 7.2, containing colonic bacterial enzymes), or 1% (v/v) phosphoric acid. The incubation periods were 2 h for SGF, 4 h for SIF, and 24 h for SCF and the phosphoric acid solution. These periods were selected to approximate the residence times of the formulation in the corresponding gastrointestinal segments. At the end of each incubation period, samples were centrifuged and the butyrate concentration in the supernatant was measured. Butyrate in the release supernatants was quantified by targeted liquid chromatography–tandem mass spectrometry for medium and short-chain fatty acids, using a triple-quadrupole linear ion trap mass spectrometer (SCIEX QTRAP 6500) equipped with an electrospray ionization source and operated in selected reaction monitoring mode. Because the release supernatants were liquid samples, they were analyzed by a procedure different from that used for the solid gastrointestinal-content and fecal samples described in Section 2.6.3, and butyrate concentrations are reported per unit volume of medium (μg/mL). Relative release was additionally calculated as a percentage of the concentration measured in 1% phosphoric acid, which was defined as the complete-release reference (100%).

#### 2.3.5 Determination of sodium butyrate drug-loading content and encapsulation efficiency

The sodium butyrate content of the CPC/SB microspheres was measured, and the drug-loading content and encapsulation efficiency were calculated as drug-loading content (%) = *M* encapsulated SB/*M* microspheres × 100% and encapsulation efficiency (%) = *M* encapsulated SB, batch/*M* initial SB, batch × 100%, where *M* encapsulated SB is the mass of sodium butyrate determined in the microsphere sample, *M* microspheres is the mass of the freeze-dried microsphere sample, *M* encapsulated SB, batch is the mass of sodium butyrate encapsulated in the microspheres obtained from one standard preparation batch, and *M* initial SB, batch is the initial mass of sodium butyrate added to that batch. Based on the measured freeze-dried product recovery of this batch (34.62 mg), the mass of encapsulated sodium butyrate was calculated to be approximately 9.43 mg, giving a drug-loading content of 27.23% (w/w) and an encapsulation efficiency of 37.70% relative to the 25 mg of sodium butyrate initially added. Batch-specific scanning electron microscopy morphology, particle-size distribution, and zeta potential were not determined for the microspheres prepared in this study.

### 2.4 Animal modeling and grouping

Thirty-five 8-week-old male C57BL/6J mice weighing 20–25 g were housed in a specific pathogen-free animal facility under controlled conditions of 22 ± 2 °C, 50 ± 10% relative humidity, and a 12-h light/dark cycle. After 1 week of acclimatization, the mice were randomly allocated to the normal-control group or the T2DM-induction cohort using a computer-generated random-number sequence.

Mice in the T2DM-induction cohort were fed a high-fat diet for 6 weeks and then received intraperitoneal injections of streptozotocin at 50 mg/kg for 3 consecutive days. Streptozotocin was freshly prepared at 10 mg/mL in sodium citrate buffer and used within 30 min. Random blood glucose was measured 7 days after the final injection. Mice with random blood glucose ≥16.7 mmol/L were considered successfully modeled and entered the intervention phase.

Successfully modeled mice were randomly allocated to six groups using a computer-generated random-number sequence (*n* = 5 per group) and received the corresponding intervention for 4 weeks. The high-fat diet was continued throughout the intervention. Together with the normal control group allocated before modeling, the study therefore comprised seven groups.

*Normal control group (CON)*: normal diet and daily gavage with an equivalent volume of normal saline (*n* = 5).

*T2DM model group (MOD)*: high-fat diet and daily gavage with an equivalent volume of normal saline (*n* = 5).

*Unloaded microsphere control group (CPC)*: high-fat diet and daily gavage with CPC microspheres suspended in normal saline at 10 mg/mL, at a formulation dose of 100 mg/(kg·day). Five mice were initially allocated to this group. One mouse died during the gavage procedure following suspected accidental aspiration and provided no endpoint samples. The remaining four mice completed the intervention and were included in the analyses (*n* = 4). No missing-value imputation was performed.

*Free sodium butyrate group (SB)*: high-fat diet and daily gavage with free sodium butyrate dissolved in normal saline at 10 mg/mL, at 100 mg/(kg·day) (*n* = 5).

*CPC/SB microsphere group (CPC/SB)*: high-fat diet and daily gavage with CPC/SB microspheres suspended in normal saline at 10 mg/mL, at a formulation dose of 100 mg/(kg·day) (*n* = 5). Based on the measured sodium butyrate loading capacity of 27.23% (w/w), this formulation dose corresponded to approximately 27.23 mg sodium butyrate/(kg·day).

*Metformin group (MET)*: high-fat diet and daily gavage with metformin dissolved in normal saline at 20 mg/mL, at 200 mg/(kg·day) (*n* = 5).

*Combination group (MET+CPC/SB)*: high-fat diet and daily gavage of metformin at 200 mg/(kg·day) plus CPC/SB microspheres at 100 mg/(kg·day) (*n* = 5).

Animals in the SB, CPC, and CPC/SB groups each received 100 mg/(kg·day) of the respective material. Based on the measured SB loading content of 27.23% (w/w), the CPC/SB dose provided approximately 27.23 mg/(kg·day) of SB and a nominal non-SB carrier fraction of 72.77 mg/(kg·day), calculated by subtraction. The free-SB and unloaded CPC groups received 100 mg/(kg·day) of SB and carrier, respectively. Thus, neither SB-equivalent nor carrier-equivalent doses were matched. In total, 35 mice were allocated to the study, and 34 completed the intervention and were included in the endpoint analyses.

### 2.5 Sample collection

#### 2.5.1 Blood glucose and body weight monitoring

Blood glucose was monitored in two ways. During the 4-week intervention, tail-vein blood was collected at a fixed time of day once weekly, and random blood glucose was measured using a SanNuo AnWen blood glucose meter with compatible test strips, to describe the time course of glycemia in each group (Figures 1E,F). The random blood glucose value obtained at the end of the intervention was also used as one of the metabolic indicators in the correlation analysis described in Section 3.6. At the end of the intervention, after a 12-h fast, fasting blood glucose was measured and used together with fasting insulin for the calculation of HOMA-IR (Section 2.6.1). Body weight was recorded at the same weekly time points and at the end of the intervention.

**Figure 1:**
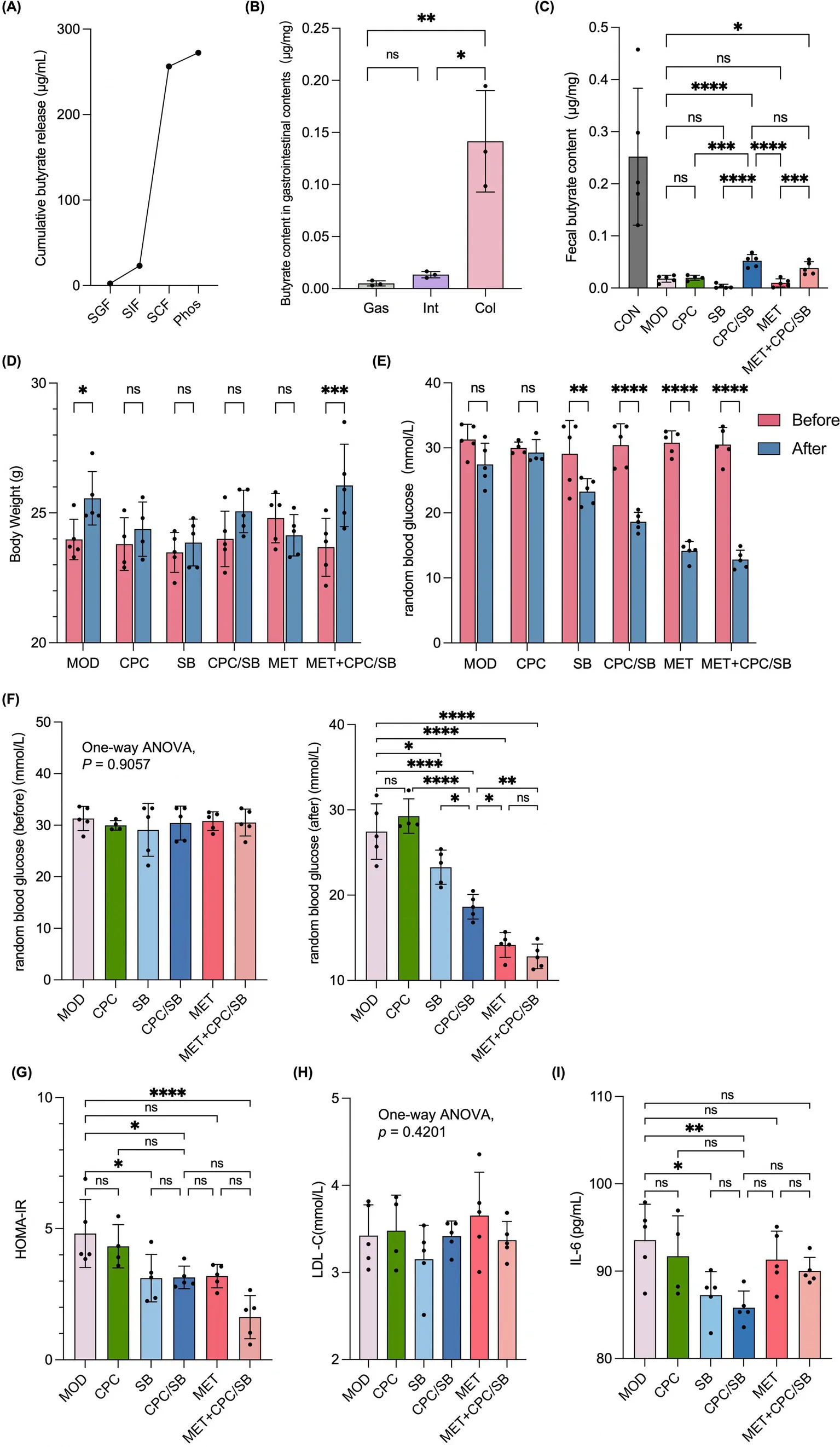
*In vitro* release characteristics of CPC/SB microspheres, gastrointestinal butyrate distribution, and metabolic outcomes in T2DM mice. **(A)** Relative butyrate release from CPC/SB microspheres under independent incubation conditions: Simulated gastric fluid (SGF, pH 1.2, 2 h), simulated intestinal fluid (SIF, pH 6.8, 4 h), simulated colonic fluid containing colonic bacterial enzymes (SCF, pH 7.2, 24 h), and 1% phosphoric acid (Phos, 24 h). The butyrate concentration measured in the phosphoric acid condition was used as the reference value (100%) for calculation of relative release. **(B)** Butyrate content in gastric (Gas), small-intestinal (Int) and colonic (Col) contents of three T2DM mice after gavage with CPC/SB microspheres. **(C)** Fecal butyrate content; the CON group is shown as a reference and was not included in the statistical comparison. **(D,E)** Body weight and random blood glucose before and after the 4-week intervention. **(F)** Random blood glucose compared between groups at baseline and at the end of the intervention. **(G–I)** HOMA-IR, serum LDL-C and serum IL-6 at the end of the intervention; HOMA-IR was calculated as fasting glucose (mmol/L) × fasting insulin (μU/mL)/22.5. Data are mean ± SD. Panels D and E comprise the six T2DM groups without CON; panels **(C,F–I)** comprise the same six T2DM groups, with CON shown in panel C as a reference. *n* = 5 mice per group, except CPC (*n* = 4); panel **(B)** included 3 mice. Panel B was analyzed by repeated-measures one-way ANOVA with Tukey’s test, panels **(C,F–I)** by ordinary one-way ANOVA with Tukey’s test, and panels **(D,E)** by a mixed-effects model with Šidák-corrected within-group comparisons. *p* values shown are multiplicity-adjusted; *p < 0.05, **p < 0.01, ***p < 0.001, ****p < 0.0001; ns, not significant. Comparisons of CPC/SB with free SB and unloaded CPC are exploratory because SB-equivalent and carrier-equivalent doses, respectively, were not matched; this applies to all figures. CON, normal control; MOD, T2DM model; CPC, CPC microspheres prepared without sodium butyrate; CPC/SB, sodium butyrate-loaded CPC microspheres; SB, free sodium butyrate; MET, metformin; MET+CPC/SB, metformin combined with CPC/SB microspheres.

#### 2.5.2 Terminal blood collection

At the end of the 4-week intervention, the mice were weighed after a 12-h fast and anesthetized by intraperitoneal injection of 2% sodium pentobarbital at 50 mg/kg. Approximately 0.8–1.0 mL of blood was collected from the orbital venous plexus into collection tubes. After standing at room temperature for 60 min, the samples were centrifuged at 3,000 rpm for 10 min, and the separated serum was stored at −80 °C until analysis.

#### 2.5.3 Euthanasia and sample collection

After blood collection, the mice were euthanized by cervical dislocation and dissected immediately. For assessment of gastrointestinal butyrate distribution, the stomach, small intestine, and colon were dissected in three T2DM mice that had received CPC/SB microspheres, and the luminal contents of each segment were collected separately into centrifuge tubes, flash-frozen in liquid nitrogen, and stored at −80 °C until analysis. Because the three segments were obtained from the same animals, these measurements were analyzed as repeated measures within mice and were not included in the between-group endpoint comparisons. Fresh fecal samples (50–100 mg per mouse) were collected from all mice at dissection, placed in sterile cryotubes, flash-frozen in liquid nitrogen, and stored at −80 °C until butyrate analysis and DNA extraction for 16S rRNA gene sequencing. Animal carcasses were disposed of in accordance with institutional biosafety regulations.

### 2.6 Metabolic parameter assays

#### 2.6.1 Blood glucose and HOMA-IR

Blood glucose was measured using a blood glucose meter and compatible test strips, as described in Section 2.5.1. Random blood glucose was used to describe the glycemic time course during the intervention and as one of the metabolic indicators in the correlation analysis, whereas fasting blood glucose was used together with fasting insulin for the calculation of HOMA-IR. HOMA-IR was calculated using the following equation: HOMA-IR = fasting glucose (mmol/L) × fasting insulin (μU/mL)/22.5. HOMA-IR was used as a surrogate index of insulin resistance.

#### 2.6.2 Serum insulin, IL-6, and LDL-C

Serum insulin, IL-6, and LDL-C were measured using commercial assay kits (catalog numbers are given in Section 2.2). Serum insulin and IL-6 were quantified by enzyme-linked immunosorbent assay and LDL-C by an enzymatic colorimetric assay. All assays were performed according to the manufacturers’ protocols, using the calibrators and standards supplied with each kit.

Serum insulin was measured using a competitive enzyme-linked immunosorbent assay. The microplate was precoated with an anti-insulin antibody, and diluted serum samples or standards were incubated with a horseradish peroxidase-labeled competing antigen. After color development with 3,3′,5,5′-tetramethylbenzidine, absorbance decreased as the insulin concentration increased because the labeled antigen competed with insulin in the sample for a limited number of antibody-binding sites.

Serum IL-6 was measured using a double-antibody sandwich enzyme-linked immunosorbent assay. The microplate was precoated with a mouse IL-6 capture antibody. Diluted serum samples or standards were incubated with a horseradish peroxidase-labeled detection antibody, followed by color development with 3,3′,5,5′-tetramethylbenzidine. Absorbance increased as the IL-6 concentration increased.

Sample preparation and the chromogenic detection steps shared by the two enzyme-linked immunosorbent assays were as follows. Serum samples were centrifuged at 3,000 rpm for 20 min at 4 °C, and the supernatants were collected. Each sample was diluted 1:10 by mixing 5 μL of serum with 45 μL of sample diluent. Samples and standards were incubated at 37 °C for 60 min, after which the plates were washed five times. Substrate solutions A and B were then added at 50 μL each, and the plates were incubated at 37 °C in the dark for 15 min. The reaction was terminated by adding 50 μL of stop solution, and the optical density at 450 nm was measured within 15 min using a SpectraMax 190 microplate reader (Molecular Devices). Concentrations were interpolated from the standard curves generated with the calibrators supplied in each kit and were multiplied by the dilution factor to obtain the values reported for undiluted serum.

Serum LDL-C was measured using a selective enzymatic colorimetric assay. Serum samples were centrifuged at 12,000 rpm for 10 min at 4 °C, and the supernatants were used for analysis. A selective surfactant was used to solubilize LDL particles. Cholesteryl esters were hydrolyzed by cholesterol esterase, and the liberated free cholesterol was oxidized by cholesterol oxidase to cholestenone and hydrogen peroxide. The hydrogen peroxide then participated in a chromogenic reaction with 4-aminoantipyrine to produce a red quinone dye. Absorbance was recorded at 546 nm at the two reading points specified in the kit protocol, and the net change was calculated as ΔA = A2 − A1. A 3.6 mmol/L LDL-C standard was used for calibration, and LDL-C concentrations are reported in mmol/L.

#### 2.6.3 Butyrate measurement in solid samples by gas chromatography–mass spectrometry

Butyrate and other short-chain fatty acids in gastrointestinal contents and feces were quantified using an Agilent 8890B gas chromatograph coupled to an Agilent 7000D triple-quadrupole mass spectrometer. Separation was performed on an HP-FFAP capillary column (30 m × 0.25 mm × 0.25 μm; Agilent J&W Scientific, Folsom, CA, USA). High-purity helium (≥99.999%) was used as the carrier gas at 1.0 mL/min. The injection volume was 1 μL with a split ratio of 10:1 and an inlet temperature of 180 °C. The oven temperature was programmed as follows: 80 °C initially, increased to 120 °C at 20 °C/min, then to 160 °C at 5 °C/min, and finally to 220 °C, held for 3 min. Mass spectrometric detection was performed in electron-impact selected-ion monitoring mode with an ion-source temperature of 230 °C, quadrupole temperature of 150 °C, transfer-line temperature of 230 °C, and electron energy of 70 eV.

2-Ethylbutyric acid (internal standard; purity ≥ 99%; Sigma-Aldrich) was prepared in n-butanol at 10 μg/mL. A mixed standard containing acetic acid, propionic acid, butyric acid, isobutyric acid, valeric acid, isovaleric acid, caproic acid, and isocaproic acid (Sigma-Aldrich; HPLC-grade n-butanol as diluent) was serially diluted to prepare a seven-point calibration curve. Although the calibration panel covered eight short-chain fatty acids, only butyrate is reported in the present study.

For solid gastrointestinal-content and fecal samples, 25 mg of sample was mixed with 500 μL of water containing 0.5% phosphoric acid and homogenized by cryogenic grinding (50 Hz, two 3-min cycles). The homogenate was sonicated for 10 min and centrifuged at 13,000 × g for 15 min at 4 °C. A 200-μL aliquot of the supernatant was mixed with 200 μL of the internal-standard solution, vortexed for 10 s, sonicated for 10 min, and centrifuged again at 13,000 × g for 5 min at 4 °C. The resulting supernatant was analyzed by GC–MS.

Data acquisition and quantification were performed using Agilent MassHunter software (version 10.0.707.0). One quality-control sample was inserted after every 5–10 study samples, and a relative standard deviation below 15% for the target analytes was considered acceptable. Butyrate concentrations in these solid samples are reported as μg/mg of wet gastrointestinal content and μg/mg of wet feces. The *in vitro* release values described in Section 2.3.4 were obtained from liquid supernatants by a different analytical procedure and are expressed per unit volume of medium (μg/mL); they are therefore not directly comparable with the per-unit-wet-mass values reported here.

### 2.7 16S rRNA sequencing

Fecal genomic DNA was extracted using the QIAamp PowerFecal Pro DNA Kit, and DNA concentration and purity were measured with a NanoDrop 2000 spectrophotometer (Thermo Fisher Scientific, Waltham, MA, United States). The V3–V4 region of the 16S rRNA gene was amplified using the primers 338F (5′-ACTCCTACGGGAGGCAGCAG-3′) and 806R (5′-GGACTACHVGGGTWTCTAAT-3′). PCR was performed in triplicate in a 20-μL reaction containing 4 μL of 5 × TransStart FastPfu buffer, 2 μL of 2.5 mM dNTPs, 0.8 μL of each primer (5 μM), 0.4 μL of TransStart FastPfu DNA polymerase, 10 ng of template DNA, and nuclease-free water to a final volume of 20 μL. The cycling programme was 95 °C for 3 min; 27 cycles of 95 °C for 30 s, 55 °C for 30 s, and 72 °C for 30 s; and a final extension at 72 °C for 10 min. Amplicons were quantified using a Synergy HTX microplate reader (BioTek, United States). Replicate amplicons were pooled and purified, and sequencing libraries were prepared using the NEXTFLEX Rapid DNA-Seq Kit (Bioo Scientific, Austin, TX, United States). Paired-end sequencing (2 × 300 bp) was performed on the Illumina NextSeq 2000 platform. After quality filtering, read merging, and DADA2 denoising, 1,349,572 high-quality sequences were retained across the 34 samples (range, 29,948–52,079 per sample; mean, 39,693), and 2,669 amplicon sequence variants (ASVs) were obtained. Raw reads have been deposited in the NCBI Sequence Read Archive under BioProject accession PRJNA1506158, and the per-sample sequencing output is available with the deposited data.

### 2.8 Bioinformatics analysis

#### 2.8.1 Data preprocessing

Paired-end reads were demultiplexed and then quality-filtered and merged using fastp and FLASH. Reads were truncated at the first position of a 50-bp sliding window whose mean quality score fell below 20; reads shorter than 50 bp after trimming or containing ambiguous bases were discarded; and paired reads were merged with a minimum overlap of 10 bp and a maximum mismatch ratio of 0.2 within the overlap region. Barcodes were required to match exactly, and up to two primer mismatches were permitted. The resulting optimized sequences were denoised using DADA2 within QIIME 2 (16) to obtain ASV representative sequences and abundance information. To equalize sequencing effort across samples, the ASV table was rarefied to an even depth of 29,948 sequences per sample, corresponding to the lowest number of sequences retained in any sample; all downstream analyses, including α-diversity, *β*-diversity, taxonomic composition, differential abundance, and functional prediction, were based on this rarefied table. No sample was excluded because of insufficient sequencing depth.

#### 2.8.2 α-Diversity analysis

Within-sample richness and diversity were assessed using the Chao1 and Shannon indices, respectively. Differences among groups were tested as described in Section 2.9.

#### 2.8.3 β-Diversity analysis

Principal coordinate analysis (PCoA) was performed based on Bray–Curtis dissimilarities at the ASV level. Between-group differences were tested by permutational multivariate analysis of variance (PERMANOVA) on the same dissimilarity matrix, as described in Section 2.9.

#### 2.8.4 Taxonomic annotation and community composition

Taxonomic annotation was performed using the SILVA 138 database, and relative abundance profiles were summarized at the phylum and genus levels. ASV representative sequences were classified using the classify-sklearn Naive Bayes classifier in QIIME 2 against the SILVA 138 database, with a confidence threshold of 0.7. Stacked bar plots were used to display the community composition of individual fecal samples, with taxa not displayed separately grouped as “Others” (Figures 2A,B). The ten genus-level taxa displayed in Figure 2B were summarized in a heatmap using the arithmetic mean of their relative abundances across mice within each group (Figure 2C). Heatma*p* values were expressed as percentages without row-wise standardization. Muribaculaceae_unclassified and Lachnospiraceae_unclassified denote taxa assigned to the respective families but not classified at the genus level. In the LEfSe results (Section 3.5.3), taxon labels retain the database prefixes used by the analysis platform (for example, norank_f__ and unclassified_f__). The Bacillota-to-Bacteroidota (F/B) ratio was calculated separately for each mouse and summarized as mean ± SD within each group (Table 1). Community composition and F/B ratios were evaluated descriptively.

**Figure 2:**
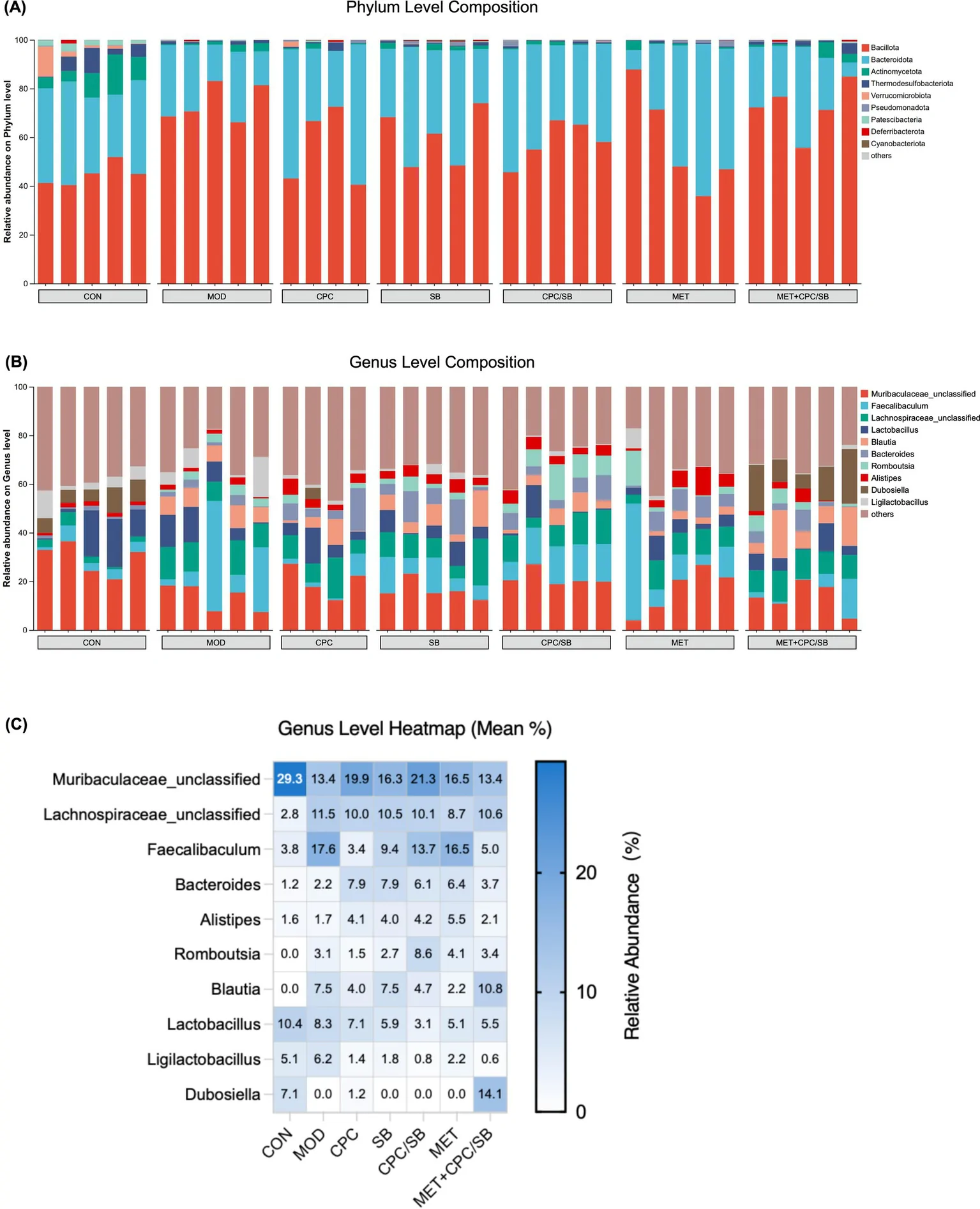
Phylum and genus-level composition of the gut microbiota. **(A)** Phylum-level relative abundance in each of the 34 fecal samples. **(B)** Relative abundance of the ten genus-level taxa with the highest mean abundance, shown per sample; taxa not displayed separately are grouped as “Others.” **(C)** Heatmap of the group mean relative abundance (%) of the same ten taxa; darker blue indicates higher abundance, and values are group means plotted without row-wise standardization. *Bacillota* and *Bacteroidota* together accounted for 80.02–97.63% of the mean community across groups. Composition is presented descriptively and was not tested for differences. Group abbreviations are as defined in Figure 1; *n* = 5 mice per group, except CPC (*n* = 4).

**Table 1:** Relative abundances of Bacillota and Bacteroidota and the Bacillota-to-Bacteroidota (F/B) ratio.

Group	Bacillota (%)	Bacteroidota (%)	F/B (mean ± SD)
CON	44.73	35.29	1.33 ± 0.43
MOD	73.97	22.85	3.73 ± 1.81
CPC	55.71	40.78	1.73 ± 1.19
SB	60.00	36.15	1.91 ± 1.00
CPC/SB	58.17	39.46	1.56 ± 0.52
MET	57.99	39.29	3.22 ± 4.41
MET+CPC/SB	72.14	22.85	5.19 ± 5.39

Bacillota and Bacteroidota values represent group mean relative abundances (%). The F/B ratio was calculated separately for each mouse by dividing the relative abundance of Bacillota by that of Bacteroidota and is presented as mean ± SD within each group. Data are summarized descriptively.

#### 2.8.5 Differential taxonomic analysis

Linear discriminant analysis effect size (LEfSe) analysis (17) was performed to identify differentially abundant taxa across five taxonomic ranks, from phylum to genus. Six separate pairwise comparisons were conducted: CON versus MOD, MOD versus CPC, MOD versus SB, MOD versus CPC/SB, MOD versus MET, and MOD versus MET+CPC/SB. Screening thresholds and the handling of multiple comparisons are described in Section 2.9. Differential-feature counts were calculated from the complete filtered results across all five taxonomic ranks. Results were visualized using linear discriminant analysis (LDA) score bar plots and taxonomic cladograms.

#### 2.8.6 Functional prediction

PICRUSt2 was used to predict microbial functional potential from 16S rRNA gene sequencing data (18). Predicted functions were mapped to the Kyoto Encyclopedia of Genes and Genomes (KEGG) database and summarized at Levels 2 and 3. At each level, the 30 categories or pathways with the highest mean predicted abundance across all samples were displayed. The Level 2 heatmap shows group mean abundances, whereas the Level 3 heatmap shows individual-sample abundances. Heatmaps display predicted abundance values without row-wise Z-score standardization. These predictions were interpreted as estimates of microbial functional potential rather than measurements of gene expression or host pathway activity.

### 2.9 Statistical analysis

Statistical analyses of the continuous outcomes in Figures 1, 3 were performed using GraphPad Prism version 10.6.0. Numerical values in the text are presented as mean ± SD unless otherwise specified. Data in Figures 1B–I are presented as mean ± SD. In Figures 3A,B, and 4E, group distributions are displayed as box plots showing the median and interquartile range, with whiskers indicating the minimum and maximum values and individual values overlaid. Taxon relative abundances reported without SD in the text or Table 1 represent group mean percentages. Community composition in Figure 2 and Table 1 and the predicted functional profiles in Figures 4B,C were summarized descriptively.

**Figure 3:**
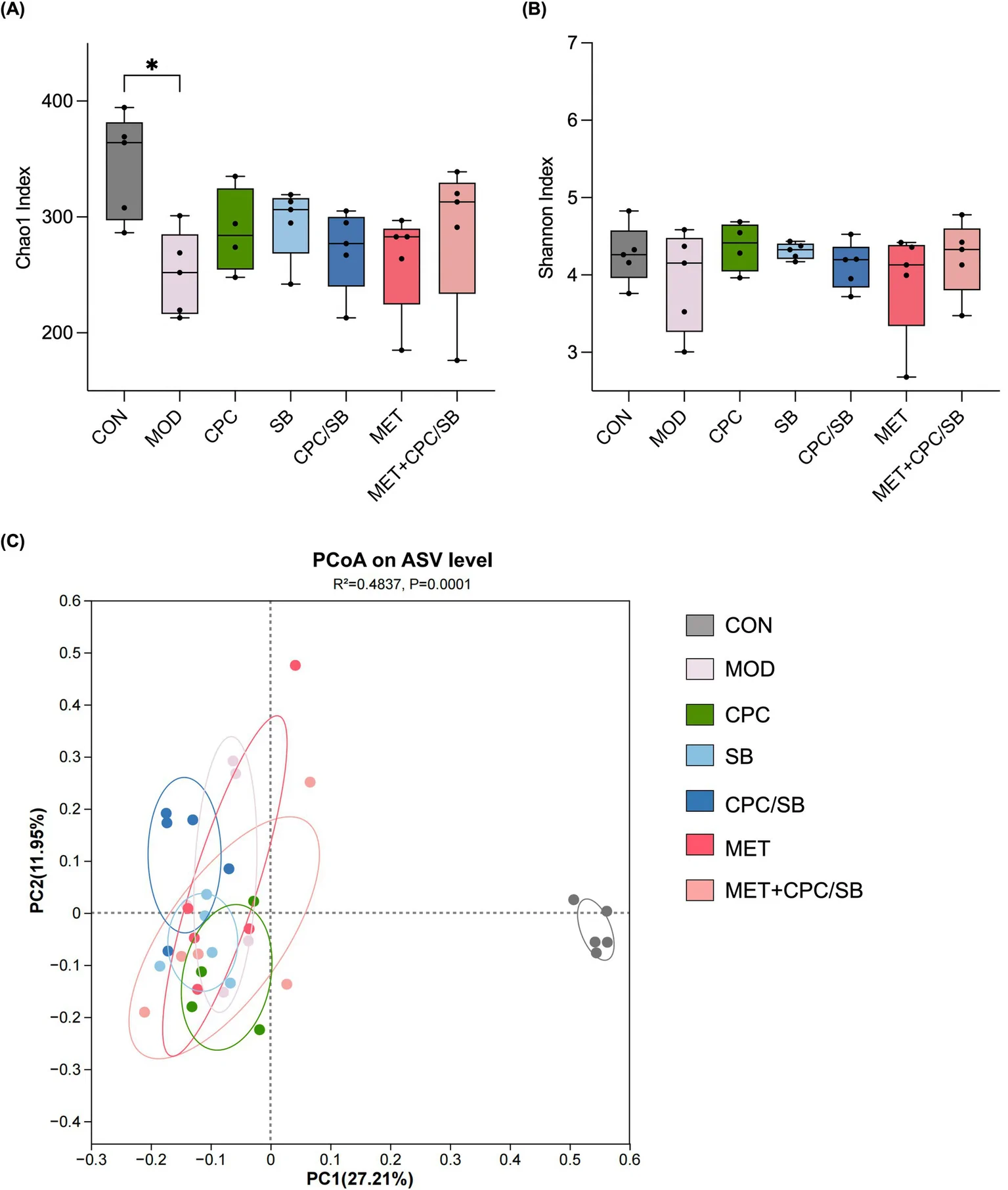
α- and β-diversity of the gut microbiota. **(A)** Chao1 richness index. **(B)** Shannon diversity index. Boxes show the median and interquartile range, whiskers the minimum and maximum, and points individual mice. **(C)** Principal coordinate analysis (PCoA) of ASV-level composition based on Bray–Curtis dissimilarities; ellipses indicate 95% confidence regions, and PC1 and PC2 explain 27.21 and 11.95% of the variation. All panels include all seven groups; *n* = 5 mice per group, except CPC (*n* = 4). Both indices were calculated from the ASV table rarefied to 29,948 sequences per sample and compared by ordinary one-way ANOVA followed by Tukey’s test; neither overall comparison reached significance [Chao1: *F* (6,27) = 2.416, *p* = 0.053; Shannon: *F* (6,27) = 0.712, *p* = 0.643], and after adjustment only the CON versus MOD contrast for Chao1 was significant. Overall differences in composition were tested by one-factor PERMANOVA with 9,999 permutations. Symbols indicating significance and group abbreviations are as defined in Figure 1.

**Figure 4:**
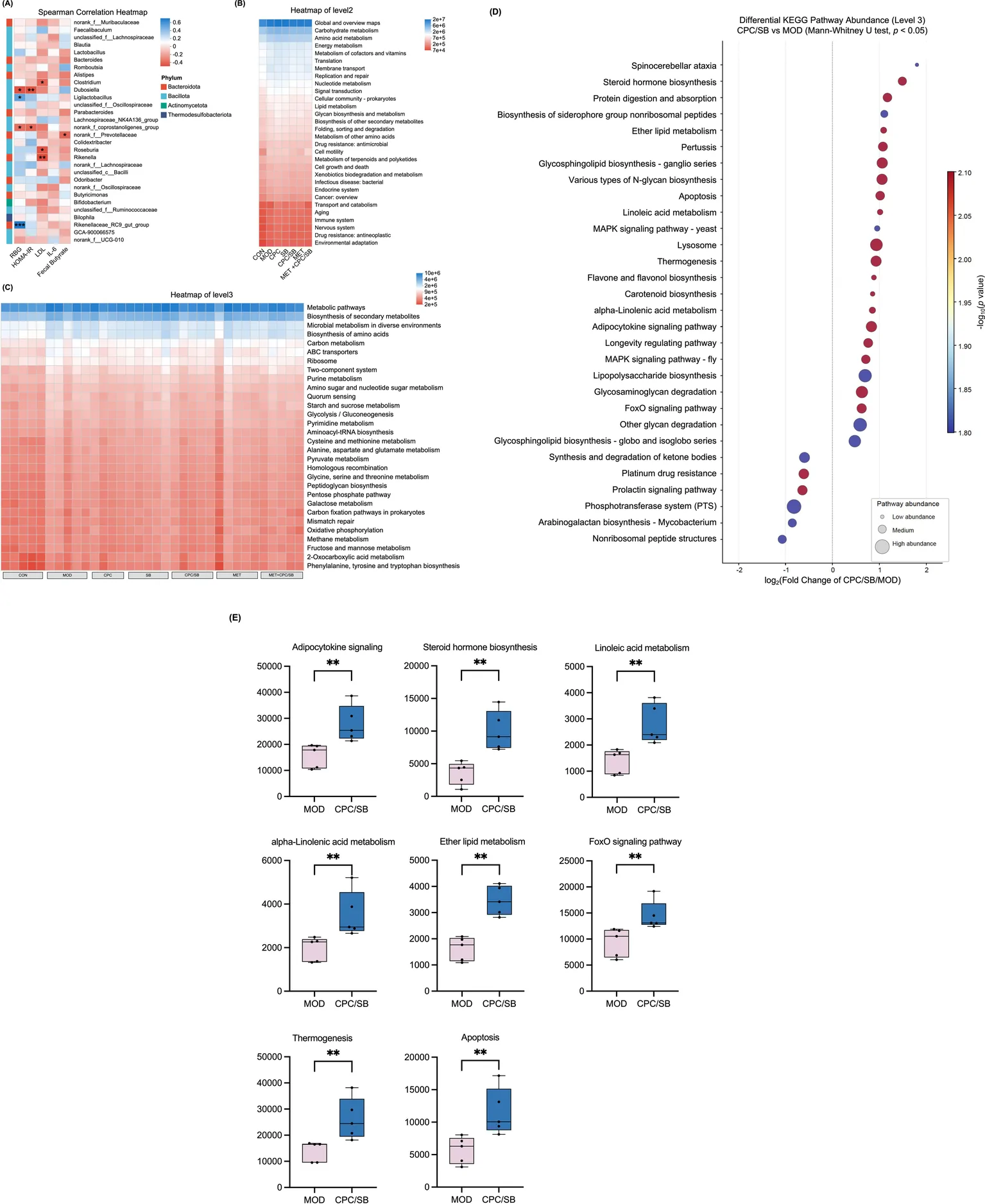
Exploratory associations between genus-level taxa and metabolic indicators, and PICRUSt2-predicted functional profiles. **(A)** Spearman correlation heatmap of the 30 genus-level taxa with the highest mean relative abundance (including the ten shown in Figure 2B) and five indicators: Random blood glucose, HOMA-IR, LDL-C, IL-6, and fecal butyrate content; blue indicates positive and red negative correlations. **(B)** Group mean predicted abundance of the 30 most abundant KEGG Level 2 categories. **(C)** Per-sample predicted abundance of the 30 most abundant KEGG Level 3 pathways. In **(B,C)**, categories and pathways were ranked by mean abundance across all samples and are plotted without row-wise standardization; darker blue indicates higher abundance. **(D)** Bubble plot of 30 of the 46 Level 3 pathways that met an unadjusted *p* < 0.05 in the MOD versus CPC/SB comparison; all 30 had an unadjusted *p* ≤ 0.0159. The horizontal axis is log2 (CPC/SB mean abundance—MOD mean abundance), bubble size is mean predicted abundance, and color is −log10 (unadjusted *p*). **(E)** Box plots of eight of these pathways; boxes show the median and interquartile range, whiskers the minimum and maximum, and points individual mice. Panels **(D,E)** include only MOD and CPC/SB; CON was excluded from panel **(A)**, and panels **(B,C)** include all seven groups. Panel **(A)** used two-sided Spearman tests and panels **(D,E)** two-sided exact Mann–Whitney U tests; Benjamini–Hochberg correction was applied across the 150 correlations and the 300 pathway tests, and no test remained significant after correction (lowest *q* = 0.0565 and *q* = 0.1190). Stars in the panels denote unadjusted *p* values (*p < 0.05, **p < 0.01, ***p < 0.001); predicted functions describe functional potential inferred from 16S rRNA gene sequences rather than measured transcript or metabolite levels. Group abbreviations are as defined in Figure 1.

Each mouse was treated as the experimental unit for the animal analyses. Endpoint analyses included five mice per group, except for the CPC group, which included four mice. For the gastrointestinal distribution experiment in Figure 1B, measurements from the stomach, small intestine, and colon of the same mice were analyzed using repeated-measures one-way ANOVA followed by Tukey’s multiple-comparisons test. For continuous outcomes measured at a single time point, including fecal butyrate, HOMA-IR, LDL-C, IL-6, Chao1, and Shannon indices, comparisons among groups were performed using ordinary one-way ANOVA followed by Tukey’s multiple-comparisons test. Random blood glucose was also compared among groups separately at baseline and at the end of the intervention using ordinary one-way ANOVA followed by Tukey’s test.

Changes in body weight and random blood glucose before and after the intervention were analyzed using mixed-effects models fitted by restricted maximum likelihood estimation. Group, time, and the group × time interaction were included as fixed effects, and repeated measurements from the same mouse were matched in the model. Šidák-corrected simple-effect comparisons were used to compare the post-intervention and pre-intervention means within each group.

A consistent parametric analysis strategy was used for the continuous outcomes in Figures 1, 3 rather than selecting tests on the basis of outcome-specific normality tests. Model residuals were examined, and homogeneity of variance was assessed using Levene’s test. For the Chao1 and Shannon indices, residual diagnostics and variance testing indicated no material departures from the assumptions of ordinary one-way ANOVA. For fecal butyrate, the variance of the CON group differed markedly from that of the model and treatment groups (Levene’s test, *p* = 0.012); therefore, CON was displayed as a physiological reference but was not included in the treatment-effect analysis in Figure 1C.

For the parametric analyses in Figures 1, 3, each analysis within a figure panel was treated as a separate family of comparisons; the baseline and week-4 analyses in Figure 1F were treated as separate families. All reported pairwise *p* values from these analyses were adjusted using the corresponding Tukey or Šidák procedure. A two-sided multiplicity-adjusted *p* < 0.05 was considered statistically significant.

PERMANOVA, LEfSe, Spearman correlation, and Mann–Whitney tests were performed on the Majorbio Cloud platform^1^. Overall differences in ASV-level community composition among the seven groups were assessed using one-factor PERMANOVA based on Bray–Curtis dissimilarities, with group as the explanatory factor and 9,999 permutations. *p* < 0.05 was used for the omnibus PERMANOVA test. LEfSe analysis included taxonomic ranks from phylum to genus and used an unadjusted Kruskal–Wallis *p* < 0.05 and a logarithmic LDA score > 3.0 as screening criteria for the six pairwise comparisons described in Section 2.8.5. Each comparison included 263 taxonomic features. Because the platform output provided exact *p* values only for features passing the nominal *p* < 0.05 threshold, unreported *p* values were assigned a value of 1.0 for a conservative multiple-testing sensitivity analysis, and the Benjamini–Hochberg procedure was applied across all 263 features within each comparison. The resulting *q* values represent conservative upper bounds. LEfSe features that did not retain *q* < 0.05 were interpreted as exploratory.

Spearman rank correlations were calculated between the relative abundances of 30 genus-level taxa and five indicators: random blood glucose, HOMA-IR, LDL-C, IL-6, and fecal butyrate content. Analyses used sample-matched data from 29 mice in the MOD and intervention groups; CON was not included. Two-sided *p* values were adjusted using the Benjamini–Hochberg (BH) procedure across all 150 correlations. These pooled correlations were interpreted as exploratory associations and not as evidence of within-group relationships or causality.

Predicted KEGG Level 3 pathway abundances were compared between MOD and CPC/SB using two-sided exact Mann–Whitney U tests (five mice per group). BH correction was applied across all 300 pathway tests. Fold change was calculated as the arithmetic mean predicted abundance in CPC/SB divided by that in MOD. Of the 300 tested pathways, 46 had an unadjusted *p* < 0.05. For display in Figure 4D, 30 of the 46 pathways were shown; these comprised the pathways with the smallest unadjusted *p* values (20 at *p* = 0.0079 and 10 at *p* = 0.0159), and the number displayed was capped at 30 for readability. The eight pathways displayed in Figure 4E were selected from the same 30 displayed pathways on the basis of their established relevance to glucose and lipid metabolism and to butyrate-related signaling; the selection was made for illustrative purposes, and all eight had an unadjusted *p* = 0.00794, the minimum attainable value for a five-versus-five exact test. Their adjusted *q* values were taken from the full 300-pathway comparison rather than recalculated for the eight displayed pathways alone. For the correlation and pathway analyses, a BH-adjusted *q* < 0.05 was used as the false discovery rate criterion. Unadjusted *p* values were retained for exploratory visualization and were distinguished from BH-adjusted *q* values; significance symbols in Figure 4 indicate unadjusted *p* values and do not denote significance after BH correction.

## 3 Results

### 3.1 *In vitro* release and gastrointestinal distribution of butyrate

The *in vitro* release experiment showed limited butyrate release from CPC/SB microspheres under simulated gastric and intestinal conditions (Figure 1A). At the specified endpoints, the measured butyrate concentrations were 2.472 μg/mL in SGF, 23.229 μg/mL in SIF, 256.433 μg/mL in SCF, and 272.305 μg/mL in the phosphoric acid reference condition. Under the independent incubation conditions, relative butyrate release was 0.91% in SGF, 8.53% in SIF, and 94.17% in SCF, using the concentration measured in the phosphoric acid condition as the reference value. Measurable butyrate release therefore increased in the order SGF < SIF < SCF.

Butyrate content also differed among gastrointestinal regions after CPC/SB microsphere administration (Figure 1B; repeated-measures one-way ANOVA, *F* (2,4) = 22.998, *p* = 0.006). The measured butyrate content was 0.0050 ± 0.0025 μg/mg in the stomach, 0.0134 ± 0.0031 μg/mg in the small intestine, and 0.1415 ± 0.0487 μg/mg in the colon. Colonic butyrate content was higher than that in the stomach and small intestine (Tukey-adjusted *p* = 0.008 and 0.010, respectively), whereas the stomach and small intestine did not differ (*p* = 0.927). The colonic value was approximately 28-fold that in the stomach and 11-fold that in the small intestine. These findings describe regional differences in total luminal butyrate after CPC/SB administration; they neither establish that intact microspheres reached the colon nor distinguish administered butyrate from endogenously produced butyrate.

### 3.2 Effects on fecal butyrate, body weight, and random blood glucose

Throughout the Results, comparisons between CPC/SB and free SB are exploratory because their SB-equivalent doses were not matched. Fecal butyrate differed among the model and intervention groups (Figure 1C; one-way ANOVA, *F* (5,23) = 22.103, *p* < 0.0001). The CPC/SB group had a higher fecal butyrate content than both the MOD group (0.0523 ± 0.0121 vs. 0.0180 ± 0.0068 μg/mg; Tukey-adjusted *p* < 0.0001) and the CPC group (0.0198 ± 0.0048 μg/mg; *p* = 0.0002). Fecal butyrate in the MET+CPC/SB group was also higher than that in the MOD group (0.0382 ± 0.0124 μg/mg; *p* = 0.015) and the CPC group (*p* = 0.048). No difference was detected between the CPC and MOD groups (*p* > 0.999). Fecal butyrate content was 0.0028 ± 0.0042 μg/mg in the SB group and 0.0100 ± 0.0079 μg/mg in the MET group; neither group differed significantly from the MOD group after adjustment for multiple comparisons.

The group × time interaction for body weight was significant (Figure 1D; *p* = 0.0037). Body weight increased from baseline in the MOD group (23.98 to 25.56 g; Šidák-adjusted *p* = 0.018) and the MET+CPC/SB group (23.68 to 26.06 g; *p* = 0.0003). No significant pre–post change was detected in the CPC, SB, CPC/SB, or MET group after multiplicity adjustment.

A significant group × time interaction was also detected for random blood glucose (Figure 1E; *p* < 0.0001). Random blood glucose decreased from baseline in the SB group (29.10 to 23.28 mmol/L; Šidák-adjusted *p* = 0.003), CPC/SB group (30.42 to 18.64 mmol/L; *p* < 0.0001), MET group (30.80 to 14.16 mmol/L; *p* < 0.0001), and MET+CPC/SB group (30.52 to 12.82 mmol/L; *p* < 0.0001). No significant pre–post change was detected in the MOD or CPC group.

Separate between-group analyses showed no difference in random blood glucose at baseline (Figure 1F; one-way ANOVA, *p* = 0.906). At the end of the intervention, the overall between-group difference was significant [*F* (5,23) = 52.973, *p* < 0.0001]. Compared with the MOD group, random blood glucose was lower in the SB (*p* = 0.037), CPC/SB (*p* < 0.0001), MET (*p* < 0.0001), and MET+CPC/SB groups (*p* < 0.0001), whereas the CPC group did not differ from the MOD group (*p* = 0.768). Blood glucose was lower in the MET and MET+CPC/SB groups than in the CPC/SB group (*p* = 0.022 and 0.002, respectively), but no difference was detected between the MET and MET+CPC/SB groups (*p* = 0.900).

### 3.3 Effects on HOMA-IR, LDL-C, and IL-6

HOMA-IR differed among the groups (Figure 1G; one-way ANOVA, *F* (5,23) = 8.477, *p* = 0.0001). Compared with the MOD group, HOMA-IR was lower in the CPC/SB group (3.14 ± 0.43 vs. 4.81 ± 1.30; Tukey-adjusted *p* = 0.046), the SB group (3.11 ± 0.91; *p* = 0.041), and the MET+CPC/SB group (1.63 ± 0.82; *p* < 0.0001). The difference between the MET and MOD groups did not reach statistical significance (3.19 ± 0.44 vs. 4.81 ± 1.30; *p* = 0.056), and the CPC group did not differ from the MOD group (4.33 ± 0.82, *n* = 4; *p* = 0.952). HOMA-IR was lower in the MET+CPC/SB group than in the CPC group (*p* = 0.001); however, it did not differ significantly from the CPC/SB or MET group after adjustment for multiple comparisons (*p* = 0.086 and 0.071, respectively).

No significant difference in serum LDL-C was detected among the model and intervention groups (Figure 1H; one-way ANOVA, *F* (5,23) = 1.036, *p* = 0.420). The corresponding group means were 3.42 ± 0.35 mmol/L in MOD, 3.48 ± 0.41 mmol/L in CPC (*n* = 4), 3.42 ± 0.17 mmol/L in CPC/SB, 3.15 ± 0.39 mmol/L in SB, 3.65 ± 0.50 mmol/L in MET, and 3.37 ± 0.22 mmol/L in MET+CPC/SB.

Serum IL-6 differed among the groups (Figure 1I; one-way ANOVA, *F* (5,23) = 4.220, *p* = 0.007). IL-6 was lower in the CPC/SB group than in the MOD group (85.82 ± 1.90 vs. 93.55 ± 4.10 pg./mL; Tukey-adjusted *p* = 0.008) and was also lower in the SB group (87.26 ± 2.68 pg./mL; *p* = 0.044). The corresponding values were 91.71 ± 4.63 pg./mL in the CPC group (*n* = 4), 91.32 ± 3.25 pg./mL in the MET group, and 90.02 ± 1.55 pg./mL in the MET+CPC/SB group; none of these groups differed significantly from the MOD group after adjustment for multiple comparisons.

### 3.4 α- and β-diversity of the gut microbiota

The Chao1 index did not reach the significance threshold among the seven groups (Figure 3A; ordinary one-way ANOVA, *F* (6,27) = 2.416, *p* = 0.053). The MOD group had a lower Chao1 index than the CON group (250.92 ± 36.25 vs. 344.46 ± 45.25; Tukey-adjusted *p* = 0.0308), consistent with reduced richness in the diabetic model. No other pairwise comparison remained significant after adjustment (all adjusted *p* ≥ 0.078), including the comparisons between the MOD group and the CPC (287.79 ± 36.71), CPC/SB (271.43 ± 35.91), SB (295.10 ± 31.05), MET (262.40 ± 44.83), and MET+CPC/SB (287.93 ± 64.69) groups.

The Shannon index did not differ significantly among the groups [Figure 3B; ordinary one-way ANOVA, *F* (6,27) = 0.712, *p* = 0.643]. The MOD group showed the lowest mean Shannon index (3.93 ± 0.65) and the CPC group the highest (4.37 ± 0.32), but no pairwise comparison was significant after Tukey adjustment (all adjusted *p* ≥ 0.777).

In both indices, no pairwise difference among the intervention groups was significant, including the exploratory comparison between CPC/SB and SB (all adjusted *p* ≥ 0.682 for Chao1 and ≥ 0.777 for Shannon). Comparisons between the CON group and the intervention groups confound disease status with treatment and are not reported as treatment effects. Thus, within-sample diversity did not differ appreciably among the intervention groups.

PCoA based on Bray–Curtis dissimilarities showed that PC1 and PC2 explained 27.21 and 11.95% of the variation, respectively (Figure 3C). The CON samples formed a distinct cluster along PC1, whereas the MOD and intervention groups showed substantial overlap. PERMANOVA with 9,999 permutations indicated an overall group-associated difference in community composition (*R*^2^ = 0.4837, *p* = 0.0001). This global test does not identify which individual group pairs differed.

### 3.5 Compositional characteristics of the gut microbiota in each group

#### 3.5.1 Phylum-level community composition

The phylum-level relative abundance profiles of the 34 fecal samples are shown in Figure 2A. Bacillota and Bacteroidota accounted for the largest proportions in all groups, together representing 80.02–97.63% of the mean community relative abundance. Compared with the CON group, the MOD group showed a higher mean relative abundance of Bacillota (73.97% vs. 44.73%) and a lower mean relative abundance of Bacteroidota (22.85% vs. 35.29%). The mean F/B ratio was 3.73 ± 1.81 in the MOD group and 1.33 ± 0.43 in the CON group (Table 1).

In the intervention groups, the mean relative abundances of Bacillota and Bacteroidota were 55.71 and 40.78% in the CPC group, 60.00 and 36.15% in the SB group, 58.17 and 39.46% in the CPC/SB group, 57.99 and 39.29% in the MET group, and 72.14 and 22.85% in the MET+CPC/SB group, respectively. The corresponding F/B ratios were 1.73 ± 1.19, 1.91 ± 1.00, 1.56 ± 0.52, 3.22 ± 4.41, and 5.19 ± 5.39. The large dispersion of the F/B ratio in the MET and MET+CPC/SB groups indicated substantial inter-individual variability. Given this variability, and because the ranking of group mean ratios did not parallel the metabolic outcomes, the F/B ratio is reported as a descriptive compositional measure. The CON group also showed higher mean relative abundances of Actinomycetota, Thermodesulfobacteriota, Verrucomicrobiota, and Patescibacteria than the other groups.

#### 3.5.2 Genus-level community composition

At the genus level, the top 10 taxa shown in Figure 2B accounted for approximately 60.55–72.62% of the mean community relative abundance across groups. The individual-sample profiles showed appreciable within-group variability. Compared with the CON group, the MOD group had a lower mean relative abundance of Muribaculaceae_unclassified (13.35 ± 5.41% vs. 29.32 ± 6.48%), and Dubosiella was not detected in the MOD samples included in this dataset (0.00 ± 0.00% vs. 7.13 ± 2.46%). In contrast, the MOD group showed higher mean relative abundances of Lachnospiraceae_unclassified (11.46 ± 2.59% vs. 2.84 ± 1.71%), Blautia (7.53 ± 1.23% vs. 0.03 ± 0.03%), and Romboutsia (3.11 ± 1.23% vs. 0.00 ± 0.00%). Faecalibaculum was also higher on average in the MOD group (17.55 ± 18.13% vs. 3.84 ± 1.93%), although marked inter-individual variability was observed.

The intervention groups displayed distinct genus-level profiles rather than a uniform shift toward the CON profile (Figures 2B,C). The CPC and CPC/SB groups had mean relative abundances of Muribaculaceae_unclassified of 19.90 and 21.27%, respectively, and mean Bacteroides abundances of 7.92 and 6.13%, respectively. Romboutsia had the highest mean relative abundance in the CPC/SB group (8.60%), whereas Alistipes had the highest mean relative abundance in the MET group (5.54%). The MET+CPC/SB group showed the highest mean relative abundances of Dubosiella (14.10%) and Blautia (10.82%). These profiles describe relative community composition rather than absolute changes in bacterial abundance. Differential-taxon analyses are presented separately in Figure 5.

**Figure 5:**
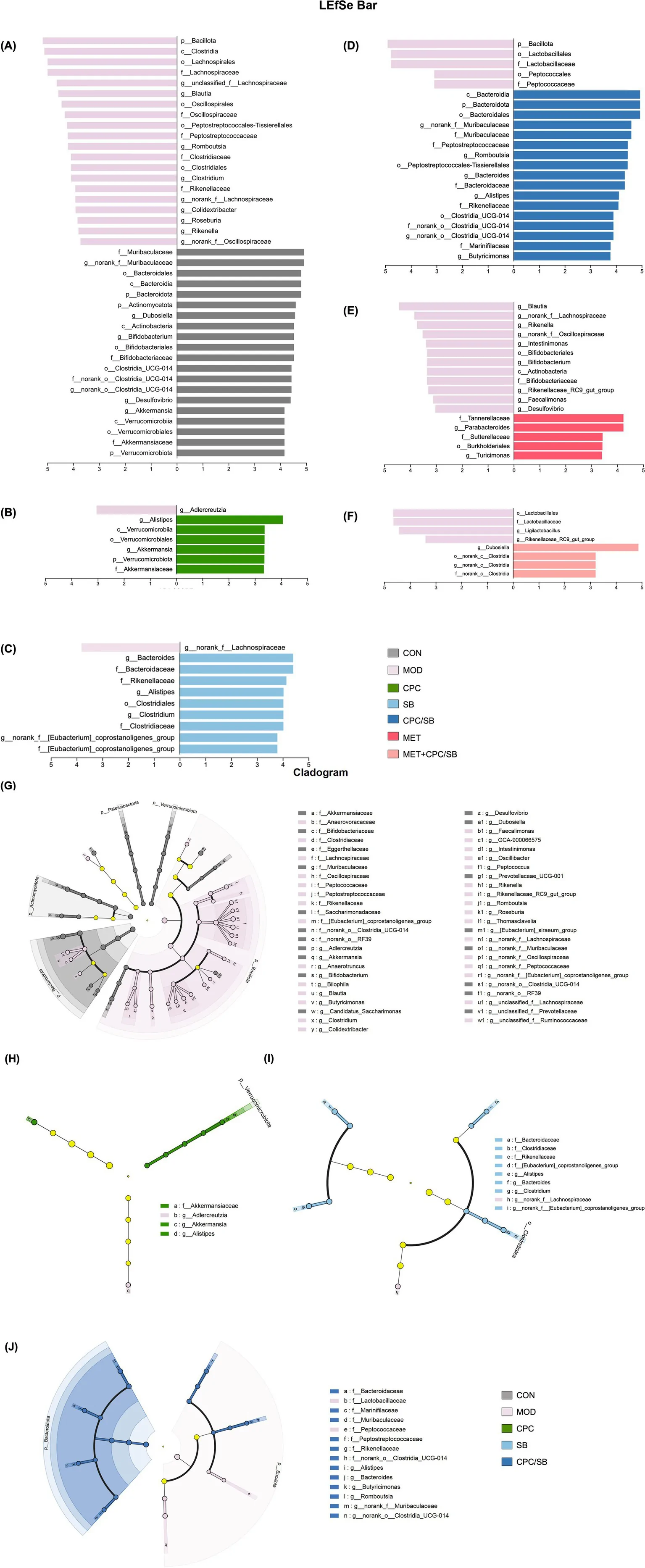
Differentially abundant taxa identified by LEfSe. Six pairwise comparisons were performed across five taxonomic ranks (phylum to genus) using Kruskal–Wallis *p* < 0.05 and a logarithmic LDA score > 3.0 as screening criteria. **(A–F)** LDA bar plots for CON versus MOD, MOD versus CPC, MOD versus SB, MOD versus CPC/SB, MOD versus MET, and MOD versus MET+CPC/SB. Panel **(A)** shows the 20 highest-scoring features enriched in each group; panels **(B,F)** show all features meeting the criteria. Bar length is the LDA score and color indicates the group in which the taxon was enriched. **(G–J)** Cladograms for CON versus MOD, MOD versus CPC, MOD versus SB, and MOD versus CPC/SB; ranks run from phylum at the centre to genus at the periphery, node size is proportional to relative abundance, colored nodes are enriched taxa and yellow nodes are taxa not identified as enriched. LEfSe *p* values are unadjusted; after conservative Benjamini–Hochberg correction across all taxa in each comparison (taxa not reported by the platform assigned *p* = 1.0), 64 of the 70 features in the CON versus MOD comparison remained significant at *q* < 0.05, whereas no feature in the MOD versus intervention comparisons did (minimum *q* = 0.17–0.55). Differential features are reported as exploratory screening results and are not used to rank treatments. Group abbreviations are as defined in Figure 1; *n* = 5 mice per group, except CPC (*n* = 4).

#### 3.5.3 LEfSe analysis of differentially abundant taxa

Using a Kruskal–Wallis *p* < 0.05 and a logarithmic LDA score > 3.0, LEfSe analysis (17) identified 70 differential taxonomic features across the phylum-to-genus ranks in the CON versus MOD comparison, including 33 enriched in CON and 37 enriched in MOD (Figures 5A,G). Taxa enriched in CON included Bacteroidota (LDA = 4.80), Muribaculaceae (LDA = 4.90), Dubosiella (LDA = 4.56), Bifidobacterium (LDA = 4.52), and Akkermansia (LDA = 4.15). Previous work has characterized Muribaculaceae as a prevalent family in the mouse gut with diverse potential for complex carbohydrate degradation (19). *Akkermansia muciniphila*, a species within the genus Akkermansia, has also been discussed in relation to T2DM and associated metabolic disorders (20). Taxa enriched in MOD included Bacillota (LDA = 5.17), Lachnospiraceae (LDA = 4.98), unclassified_f__Lachnospiraceae (LDA = 4.63), and Blautia (LDA = 4.57). Unadjusted Kruskal–Wallis *p* values for the 70 features ranged from 0.005 to 0.047.

The MOD versus CPC comparison identified seven differential features, with six enriched in CPC and one in MOD (Figures 5B,H). Alistipes (LDA = 4.06) and Akkermansia (LDA = 3.36) were enriched in CPC, whereas Adlercreutzia (LDA = 3.04) was enriched in MOD; unadjusted *p* values for these seven features ranged from 0.027 to 0.029. The MOD versus SB comparison identified ten differential features, including nine enriched in SB and one in MOD (Figures 5C,I). Bacteroides (LDA = 4.40), Alistipes (LDA = 4.02), and Clostridium (LDA = 4.02) were enriched in SB, whereas the genus-level taxon norank_f__Lachnospiraceae (LDA = 3.81) was enriched in MOD; unadjusted *p* values for these ten features ranged from 0.009 to 0.047.

The MOD versus CPC/SB comparison identified 22 differential features, including 17 enriched in CPC/SB and five in MOD (Figures 5D,J). Taxa enriched in CPC/SB included Bacteroidota (LDA = 4.92), Muribaculaceae (LDA = 4.58), Romboutsia (LDA = 4.44), Bacteroides (LDA = 4.33), Alistipes (LDA = 4.10), and Butyricimonas (LDA = 3.77). Bacillota (LDA = 4.91), Lactobacillales (LDA = 4.77), and Peptococcales (LDA = 3.08) were enriched in MOD. Unadjusted *p* values for these 22 features ranged from 0.009 to 0.047.

The MOD versus MET comparison identified 17 differential features, with five enriched in MET and 12 in MOD (Figure 5E). Parabacteroides (LDA = 4.23) and Turicimonas (LDA = 3.40) were enriched in MET, whereas Blautia (LDA = 4.43) was enriched in MOD; unadjusted *p* values for these 17 features ranged from 0.009 to 0.047. The MOD versus MET+CPC/SB comparison identified eight differential features, with four enriched in each group (Figure 5F). Dubosiella showed the highest LDA score among the features enriched in MET+CPC/SB (LDA = 4.86), and the order-level taxon norank_c__Clostridia was also enriched in this group (LDA = 3.20). Ligilactobacillus (LDA = 4.43) and Rikenellaceae_RC9_gut_group (LDA = 3.39) were enriched in MOD; unadjusted *p* values for these eight features ranged from 0.005 to 0.047.

In a conservative BH sensitivity analysis across all 263 tested taxonomic features within each comparison, in which features without a reported *p* value in the LEfSe output were assigned *p* = 1.0, 64 of the 70 LEfSe features identified between CON and MOD retained *q* < 0.05. None of the LEfSe features identified in the five MOD-versus-intervention comparisons retained *q* < 0.05.

### 3.6 Associations between genus-level taxa and metabolic indicators

In the present study, associations between genus-level relative abundances and metabolic indicators were explored using Spearman correlation analysis in 29 mice from the MOD and intervention groups (Figure 4A).

For glucose metabolism, Rikenellaceae_RC9_gut_group showed the strongest positive correlation with random blood glucose (RBG; *ρ* = 0.616, *p* = 0.000377, *q* = 0.0565). Ligilactobacillus was also positively correlated with RBG (*ρ* = 0.451, *p* = 0.0142). Dubosiella was negatively correlated with RBG (*ρ* = −0.464, *p* = 0.0112) and HOMA-IR (*ρ* = −0.473, *p* = 0.0095). The genus-level taxon norank_f__[Eubacterium]_coprostanoligenes_group also showed negative correlations with RBG (*ρ* = −0.372, *p* = 0.0471) and HOMA-IR (*ρ* = −0.399, *p* = 0.0322).

For lipid metabolism, Rikenella (*ρ* = −0.486, *p* = 0.0076), Roseburia (*ρ* = −0.458, *p* = 0.0124), and Clostridium (*ρ* = −0.409, *p* = 0.0274) were negatively correlated with LDL-C at the unadjusted threshold. No correlations with IL-6 met the unadjusted *p* < 0.05 threshold. For fecal butyrate content, norank_f__Prevotellaceae showed a negative correlation (*ρ* = −0.410, *p* = 0.0270).

Ten of the 150 correlations met the unadjusted *p* < 0.05 threshold, but none remained significant after BH correction; the lowest *q* value was 0.0565.

### 3.7 Predicted functional profiles of the gut microbiota

PICRUSt2-based functional prediction was used to characterize the potential functional profiles of the gut microbiota (18). At KEGG Level 2, the most abundant predicted categories included “Global and overview maps,” “Carbohydrate metabolism,” and “Amino acid metabolism” (Figure 4B). At Level 3, the individual-sample heatmap displayed the 30 pathways with the highest mean predicted abundance across samples, including “Metabolic pathways,” “Biosynthesis of secondary metabolites,” “Microbial metabolism in diverse environments,” and “Biosynthesis of amino acids” (Figure 4C). These heatmaps provide descriptive summaries of predicted functional composition. The 30 Level 3 pathways displayed in Figure 4C were selected solely on the basis of mean predicted abundance across all samples and are therefore distinct from the 30 pathways displayed in Figure 4D, which were selected from among those differing between MOD and CPC/SB.

In the MOD versus CPC/SB comparison, 46 of the 300 tested Level 3 pathways met the unadjusted *p* < 0.05 threshold, and 30 of these 46 pathways were displayed in Figure 4D. None of the 300 pathways met the BH-adjusted *q* < 0.05 criterion; the lowest *q* value was 0.1190. Pathways with higher predicted abundance in CPC/SB included linoleic acid metabolism, alpha-linolenic acid metabolism, ether lipid metabolism, FoxO signaling, and adipocytokine signaling. Among the 46 pathways, those with lower predicted abundance in CPC/SB included the phosphotransferase system (the principal bacterial machinery for carbohydrate uptake), nonribosomal peptide structures, and synthesis and degradation of ketone bodies.

Eight pathways from this comparison were displayed as individual-value box plots (Figure 4E). Their mean predicted abundances were higher in CPC/SB than in MOD: adipocytokine signaling pathway (FC = 1.78), steroid hormone biosynthesis (FC = 2.81), linoleic acid metabolism (FC = 2.02), alpha-linolenic acid metabolism (FC = 1.80), ether lipid metabolism (FC = 2.13), FoxO signaling pathway (FC = 1.54), thermogenesis (FC = 1.90), and apoptosis (FC = 2.02). All eight yielded an unadjusted *p* = 0.00794 and a BH-adjusted *q* = 0.1190. The unadjusted *p* value of 0.00794 is the smallest attainable with five mice per group in a Mann–Whitney test and reflects complete separation of the two groups.

## 4 Discussion

This study shows that CPC/SB microspheres improved several metabolically relevant outcomes in mice with high-fat diet and streptozotocin-induced diabetes. CPC/SB treatment increased fecal butyrate, lowered random blood glucose and HOMA-IR, and reduced circulating IL-6 compared with the untreated diabetic group. The glycemic improvement was accompanied by exploratory taxonomic and predicted functional findings, while LDL-C and α-diversity remained unchanged. These findings extend the evaluation of this formulation from intestinal barrier injury models to experimental diabetes (15).

The *in vitro* release pattern is consistent with the intended colon-directed design and with the findings reported for the original CPC/SB formulation in intestinal injury models (15). CPC/SB microspheres released little butyrate under simulated gastric conditions, a modest amount under simulated intestinal conditions, and most of the measurable butyrate under the simulated colonic condition. In treated mice, butyrate content was substantially higher in the colon than in the stomach or small intestine.

Fecal butyrate provides an additional measure of intestinal butyrate availability. It represents the fraction remaining after microbial production, epithelial uptake, metabolism and intestinal transit, and colonic short-chain fatty acids are extensively utilized by the epithelium and liver before reaching the systemic circulation (21). In this study, CPC/SB increased fecal butyrate relative to the model group, whereas free SB lowered blood glucose and HOMA-IR without a significant increase in fecal butyrate. Rapid proximal absorption of unencapsulated butyrate (11) offers one possible explanation for the latter observation. Comparisons between the CPC/SB and free-SB regimens are exploratory because their SB-equivalent doses were not matched.

Several established actions of butyrate provide plausible links between distal intestinal exposure and the metabolic phenotype observed here. Butyrate improves insulin responsiveness and increases energy expenditure in diet-induced obese mice (22), stimulates enteroendocrine GLP-1 secretion through FFAR2 (6), supports epithelial barrier function through hypoxia-inducible factor-dependent signaling (23) and in monolayer models of the intestinal barrier (24), and inhibits histone deacetylases with downstream effects on immune and metabolic gene expression (5). These actions converge on pathways governing glucose handling, epithelial integrity and systemic inflammatory tone, and buttressed barrier function limits the translocation of bacterial products that sustain low-grade inflammation (8, 9). In the present study, the parallel reductions in blood glucose, HOMA-IR and IL-6 form a coherent response compatible with this framework. The IL-6 result is particularly relevant because chronic low-grade inflammation contributes to insulin resistance and metabolic dysfunction in diet-induced diabetes.

The metabolic response was selective rather than uniform. CPC/SB produced clear changes in blood glucose and in the fasting glucose–insulin relationship, whereas LDL-C was unchanged and body weight was not reduced during the four-week intervention. This pattern indicates that the early response to CPC/SB centers on glycemic regulation and inflammatory tone, whereas changes in circulating lipids and adiposity may require longer exposure or may not be major components of its action in this model; the effects of butyrate on lipid profiles and body weight have been inconsistent across studies (12, 25). HOMA-IR remains a surrogate index in streptozotocin-treated mice, and its reduction alongside random blood glucose strengthens the evidence for improved glucose homeostasis. It should also be noted that metformin and metformin combined with CPC/SB lowered blood glucose more than CPC/SB alone (*p* = 0.022 and 0.002, respectively), so CPC/SB improved glycemia relative to the untreated model without matching the effect of metformin in this model.

The microbiota findings suggest differences in taxonomic profiles without a detectable increase in community diversity. Neither Chao1 nor Shannon diversity differed significantly among the intervention groups. At the community level, normal-control samples were clearly separated from the diabetic groups, whereas the intervention groups occupied partially overlapping regions of the ordination space. At finer resolution, CPC/SB was characterized by higher relative abundances of Bacteroidota, Muribaculaceae, Romboutsia, Bacteroides, Alistipes and Butyricimonas. Several of these taxa degrade complex carbohydrates or participate in cross-feeding networks that support short-chain fatty-acid metabolism (3, 4); Muribaculaceae in particular is a prominent mouse-gut family with diverse carbohydrate-utilization potential (19), and members of the phylum Bacteroidota have been reported to be altered in human type 2 diabetes (2). The unloaded carrier group also showed exploratory enrichment of Akkermansia and Alistipes. Given these exploratory findings and previous reports on chitosan materials (13, 14, 20), an independent microbiota-modulating effect of the carrier cannot be excluded. The absence of significant metabolic changes in the unloaded CPC group is therefore not interpreted as evidence that the carrier is inactive.

The genus-level patterns further show that metabolic benefit was associated with distinct microbial configurations rather than restoration of a single universal “healthy” profile. Dubosiella was most abundant in the metformin plus CPC/SB group and showed inverse correlations with blood glucose and HOMA-IR in the pooled exploratory analysis; experimental work on *D. newyorkensis* has demonstrated immunomodulatory activity in colitis (26), providing a rationale for investigating this genus in metabolic inflammation. Blautia also varied across treatment groups—it was enriched in the model group relative to normal controls yet most abundant in the combination group—and this genus is functionally heterogeneous, with *B. coccoides* shown to support colonic mucus function through short-chain fatty-acid production under low-fiber conditions (10). Together with the Muribaculaceae, Alistipes, Butyricimonas and Romboutsia signals, these taxa define a biologically plausible set of candidates for mechanistic follow-up. Because the intervention-associated LEfSe and correlation findings did not remain significant after false-discovery-rate correction, they are best used to prioritize targeted experiments rather than to assign the observed metabolic effect to any single genus.

The predicted functional profiles reinforce this hypothesis-generating picture. Compared with the model group, CPC/SB showed higher predicted abundance of pathways related to linoleic and alpha-linolenic acid metabolism, ether lipid metabolism, adipocytokine signaling, FoxO signaling and thermogenesis, and lower predicted abundance of the phosphotransferase system, nonribosomal peptide structures, and ketone body turnover. These functions align with the observed improvement in glucose regulation and point to microbial metabolic capacities that may influence host energy and lipid signaling. PICRUSt2 infers functional potential from marker-gene profiles and reference genomes (18). The convergence of several predicted functions on metabolic regulation therefore provides a basis for subsequent shotgun metagenomic, metabolomic and host-tissue analyses.

The metformin groups provide an informative comparison. Metformin alone produced the greatest glucose lowering among the single treatments, whereas the combination group showed the lowest numerical blood glucose and HOMA-IR values overall. The combination also produced a distinct microbial profile, including marked enrichment of Dubosiella and Blautia. Metformin is known to alter the gut microbiome in people with treatment-naive type 2 diabetes, and microbiota-transfer experiments support a contribution of these changes to its metabolic effects (27, 28); metformin also acts through AMP-activated protein kinase (29) and modifies microbial metabolite fractions in patients with type 2 diabetes (30). Microbial metabolites beyond butyrate are equally relevant in this setting, since microbiota-derived hydrogen sulfide has been shown to impair host metabolism by lowering GLP-1 (31). In the present study the combination did not differ significantly from metformin alone for blood glucose or HOMA-IR, so the data support compatibility and potential complementary activity rather than a definitive interaction; a factorial study designed and powered to test the treatment interaction would be appropriate for evaluating add-on benefit.

Several limitations should guide interpretation and future work. The study used small groups of male mice and a four-week intervention in the high-fat diet and streptozotocin model. As detailed in Section 2.4, the unmatched SB-equivalent and carrier-equivalent doses prevent separation of SB, carrier, and delivery-related effects. The unloaded CPC group had only four evaluable mice after one death during gavage, limiting the precision of its estimates. Only one CPC/SB dose was tested, so dose dependence and an optimal dose remain undetermined. The present study does not provide a dose recommendation. Batch-specific physicochemical characterization was incomplete, and independent *in vitro* incubations and unlabeled luminal butyrate measurements do not establish *in vivo* targeting efficiency. Only IL-6 was measured as a circulating inflammatory marker; baseline microbiota samples and cage-effect analyses were unavailable, and microbial functions were inferred from 16S rRNA profiles. Subsequent studies require matched-dose controls, multiple CPC/SB dose levels, complete microsphere characterization, and labeled-drug or particle tracking, alongside longitudinal microbiota sampling and direct measurements of glucose tolerance, intestinal barrier function, and tissue insulin signaling. Microbiota-transfer or depletion experiments would help determine whether microbial changes contribute to the metabolic response.

Clinical translation will depend on metabolic context and on the ability of the formulation to reproduce distal intestinal exposure in humans. Oral butyrate has shown differential metabolic effects in lean participants and in participants with metabolic syndrome (32), emphasizing the importance of patient phenotype and delivery strategy. The present results provide a preclinical rationale for further evaluation of CPC/SB microspheres: treatment increased fecal butyrate, improved glycemic indices, and reduced circulating IL-6 relative to the diabetic model group, alongside exploratory findings on microbial composition and predicted function. These findings support further evaluation of the complete formulation, but SB, carrier, and delivery-related effects cannot be separated, and its *in vivo* targeting efficiency remains unverified.

## Data Availability

The data analyzed in this study are available in the NCBI repository (https://www.ncbi.nlm.nih.gov) under BioProject accession number PRJNA1506158. Additional data are available from the corresponding author upon reasonable request.

## Footnotes

1
https://cloud.majorbio.com
